# The perioperative management of geriatric patients in transplantation surgery—clinical, immunological, and translational considerations

**DOI:** 10.3389/frtra.2025.1566466

**Published:** 2025-09-26

**Authors:** Leonard Knoedler, Sam Boroumand, Christopher A. Hinze, Samuel Knoedler, Alexandre G. Lellouch, Bhagvat J. Maheta, Jasper Iske, Adriana C. Panayi

**Affiliations:** ^1^ Division of Plastic and Reconstructive Surgery, Cedars-Sinai Medical Center, Los Angeles, CA, United States; ^2^ Division of Plastic and Reconstructive Surgery, Department of Surgery, Yale New Haven Hospital, Yale School of Medicine, New Haven, CT, United States; ^3^ Hannover Medical School, Department of Respiratory Medicine Hannover Medical School, Department of Respiratory Medicine and Infectious Diseases, Hannover, Germany; ^4^ Biomedical Research in Endstage and Obstructive Lung Disease Hannover (BREATH), Member of the German Center for Lung Research (DZL), Hannover, Germany; ^5^ Vascularized Composite Allotransplantation Laboratory, Center for Transplantation Sciences, Massachusetts General Hospital, Harvard Medical School, Boston, MA, United States; ^6^ Université Paris Cité, Inserm, The Paris Cardiovascular Research Center, Team Endotheliopathy and Hemostasis Disorders, Paris, France; ^7^ Hematology Department, AP-HP, Hôpital Européen Georges Pompidou, Paris, France; ^8^ Department of Surgery, Northwestern University Feinberg School of Medicine, Chicago, IL, United States; ^9^ Department of Cardiothoracic and Vascular Surgery, Deutsches Herzzentrum der Charité, Berlin, Germany; ^10^ Charité Universitätsmedizin Berlin, Berlin, Germany; ^11^ Division of Plastic Surgery, Department of Surgery, Brigham and Women’s Hospital, Harvard Medical School, Boston, MA, United States

**Keywords:** geriatrics, frailty, solid organ transplant, SOT, vascularized composite allotransplantation, VCA

## Abstract

Transplant surgery encompasses two primary branches: solid organ transplantation (SOT) and vascularized composite allotransplantation (VCA). As the global population ages, elderly transplant patients become a more pressing clinical challenge. Elderly transplant recipients require specialized care that addresses their unique needs, including increased comorbidities and frailty. Despite the growing recognition of these challenges, there is a paucity of studies that synthesize the current knowledge on this patient cohort, from immunological changes over translational challenges to tailored clinical care. This review highlights the individual needs of elderly transplant patients, emphasizing the importance of understanding their clinical profiles to develop specialized perioperative management strategies. The clinical need for tailored therapeutic concepts contrasts with the current lack of established, integrated care models specifically designed for older adults undergoing SOT and VCA. Overall, future research is warranted to provide individualized and cross-disciplinary care models for aging transplant patients and broaden the access to transplant surgery for this patient population.

## Introduction

Transplant surgery in geriatric patients (65+ age) has seen a significant rise over the past decades (1–4). Solid organ transplantation (SOT) is a life-saving surgery for patients presenting with end-stage solid organ failure. Over the past decades, the number of SOTs in elderly patients has continuously grown, currently amounting to 14% of all SOTs performed in the U.S. (5, 6). Besides SOT, vascularized composite allotransplantation (VCA) represents a dynamic branch of reconstructive surgery to restore form and functionality in patients with severe trauma and irreversible tissue loss (7). VCA surgery involves the transplantation of diverse tissue types, encompassing skin, mucosa, blood and lymphatic vessels, muscle, and bone, paving a novel treatment pathway for complex reconstructive cases (8–11). Paralleling SOT, geriatric patients above the age of 65 also account for ∼14% of VCA procedures, with the 50–65 age group comprising ∼28% (5, 12). Interestingly, previous research has revealed a lower incidence of rejection episodes, however, an increased risk of transplant failure in both elderly SOT and VCA patients (13).

These postoperative challenges and the increasing number of SOT and VCA surgeries in elderly patients highlight the pressing need for targeted strategies to optimize perioperative care. However, there is a paucity of research condensing the clinical, immunological, and translational knowledge of elderly transplant patients. This research gap leaves untapped potential to advance perioperative management and improve postoperative outcomes in geriatric transplant surgery. To bridge this gap, we aimed to provide a holistic overview of different types of SOT (liver, kidney, heart, lung) and VCA surgery (face, hand, penis, uterus, abdominal wall) in geriatric populations.

## Solid organ transplantation

### Age-specific clinical considerations and common risk factors of postoperative complications

The notably lower percentage of SOT within the geriatric population reflects the higher risk of perioperative complications that can occur compared to younger patients. SOT is a unique surgical consideration for geriatric patients as patients undergoing organ transplantation surgery are often seriously ill and the surgery itself can result in significant hemodynamic shifts that may impact geriatric patients significantly more (13). A study on kidney transplantation that looked at short-term outcomes in the context of primary non-function, delayed graft function, length of hospital stay, and death during initial hospitalization found that there was no statistically significant difference in geriatric patients compared to individuals younger than 65 (14, 15). However, individuals 65 years and older were found to have a 39% increased mortality rate over a period of 5 years compared to their younger counterparts (14, 15). Additionally, a study based in Korea detected a higher incidence of early post-transplant infection after kidney transplantation, mainly affecting the urinary tract associated with rejection, graft loss and all-cause mortality in patients older than 60, suggesting age is a prominent variable influencing the long-term success of SOT (16).

### Diabetes mellitus type II

The landscape of age-related clinical considerations in geriatric SOT patients is multifactorial and encompasses a range of comorbidities with higher prevalence in older people (17–19). Comorbidities such as hypertension, diabetes, osteoarthritis, dementia, chronic kidney failure and a history of previous cardiac surgery can introduce additional degrees of medical complexity to the transplantation process. In geriatric patients undergoing kidney transplantation, diabetes was highly prevalent, representing the second most common comorbidity (65.5%) and a leading cause of kidney failure (49.1%). Univariate analysis revealed that diabetes was associated with a more than doubled risk of mortality. Furthermore, a significantly higher proportion of patients deemed less suitable for transplant had diabetes (80.5% vs. 53.2%) compared to those considered good candidates, highlighting the role of diabetes as a comorbidity in transplant selection (20). Stepanova et al. recently performed a large register analysis that identified growing numbers of patients with diabetes type II pre-transplant negatively affecting post-transplant mortality in heart, lung, liver and kidney transplantation as well as graft lost after kidney transplantation alone. Sociodemographic analysis highlights a significantly higher mean age of patients suffering from diabetes than with no diabetes emphasizing a considerable negative impact on post-transplant outcome (21).

### Cardiovascular comorbidities

In addition to diabetes, cardiovascular risk factors, which are more common in older patients, also impact SOT. Agarwal et al. also showed that kidney transplant patients, with a mean age of 52.7, with an average systolic blood pressure (SBP) in the 121–130 mm Hg range had a probability of five-year graft survival after transplantation that was 2% higher compared to patients with higher blood pressures. They noted that recipients with SBP's greater than 130 mm Hg had higher prevalence of type 2 diabetes mellitus, obesity, sleep apnea, and coronary artery disease (22). On the other hand, studies in different surgery types have shown no impact on surgical outcomes in older age groups. Page et al. found that history of myocardial infarction and inhalation therapy, for example, did not have a statistically significant difference on complication rates in gastrointestinal surgery compared to patients without these age-associated comorbidities (23). Geriatric patients undergoing surgery, particularly those with cardiovascular disease, face an elevated risk of both bleeding and thrombosis; thus, a careful balance must be maintained. To mitigate intraoperative bleeding complications, anticoagulation is typically held prior to the procedure, and anticoagulation is typically restarted immediately postoperatively to minimize risk of clot formation based on patient factors (24). Overall, however, the higher prevalence of comorbidities in older patients and the observed correlation with SOT outcomes demonstrate that age-associated comorbidities significantly contribute to SOT outcomes specifically, and need to be included in follow-up care.

### The need for prehabilitation and the role of frailty

Frailty is an additional age-associated risk factor that may contribute to the difference in SOT outcomes between older and younger patients. Assessed through tools like the Groningen Frailty Index (GFI) or the five-item modified frailty index (mFI-5), frailty is a geriatric syndrome associated with less resilience to stress and a decrease in physiological reserves (13, 18, 19, 25, 26). In the growing population of elderly patients on the kidney transplant waitlist, sarcopenia, characterized by loss of muscle mass and strength, represents a significant and often overlooked factor that can negatively impact post-transplant outcomes, including graft survival and overall mortality (27). Patients with a higher GFI presented with a twofold risk of mortality following SOT and with a 61% higher chance of hospital readmission within the first month following SOT (28, 29). This finding can also be extrapolated to kidney transplantation, as geriatric patients have been shown to have an 80% higher risk of delayed recovery following the procedure (30, 31). Frailty also caused increased mortality as well as longer in-hospital stay after heart transplantation (32). Varughese et al. created the solid organ transplant frailty index (FI) specifically for transplant surgery screening, which is a tool derived from routinely collected patient data during transplant candidacy evaluations, based on the cumulative deficits model, and has been shown to help assess frailty and predict adverse outcomes, including waitlist mortality and post-transplant survival (33). Frailty indexes such as the solid organ transplant FI can help with donor selection in elderly patients, especially with expanded criteria donor (ECD) organs potentially increasing surgical risks in this population (34). Nevertheless, while higher frailty, as determined by frailty indexes, increases the risk of complications in transplant surgery, the potential benefits of a transplant may outweigh these risks in carefully selected cases, thus advocating for personalized care models and shared decision making with the patients.

### Cognitive impairments

The cognitive abilities of a patient are also affected by transplantation. Dementia or other cognitive disorders are less favorable preoperative conditions for transplantation because this can potentially lead to postoperative delirium (35). Elderly patients have a higher risk of experiencing postoperative dementia or delirium compared to younger individuals (35). In fact, this condition can manifest in approximately 10%–50% of geriatric patients following surgery and can occur in older patients even when they appear outwardly healthy (36). In a study that monitored patients after kidney transplantation, postoperative delirium was observed in 13.8% of patients aged 75 and older compared to 2% of recipients aged 18–49 years old (37). Special considerations must be made for the administration of certain pain medications, such as meperidine, that can lead to delirium and cognitive decline in geriatric patients (38). The higher prevalence of preoperative dementia and postoperative delirium in geriatric patients highlights an additional age-related factor that must be considered in the context of SOT.

### Immunological shifts and their clinical impact in aging transplant patients

#### Adaptive immunity

Immunosenescence refers to the gradual decline of the immune system in older individuals (39). Due to the complex, age-related process of thymic involution, the amount of naïve T cells decreases, resulting in peripheral expansion of memory T cells (40–42). The decline in naïve cells serves as a crucial indicator of immune system frailty in older patients and contributes to the overall vulnerability of older individuals to infections and chronic diseases. The accumulation of memory T cells provides a protective mechanism against previously encountered pathogens, while the decline in naïve T cells heightens susceptibility to newly emerging pathogens (43). Of note, Naylor et al. detected a dramatic loss of T cell receptor diversity in patients aged between 65 and 75 years, potentially responsible for attenuated T cell responses to novel antigens (44).

With increasing age, the decline of naïve T cells is accompanied by reduced expression of the costimulatory factor CD28 in both CD4+ and CD8+ cells, as well as decreased cytotoxic efficacy in CD8+ and natural killer cells (45, 46). Given that aging is associated with an accumulation of mitochondrial stress and a lack of telomerase activity to resolve stressor impacts on DNA structure, T lymphocytes gradually enter an immunosenescent state and accumulate as memory T cells (47, 48). As older patients approach an immunosenescent state, this condition can be effectively detected in T cells by using the marker TIGIT, which is elevated in T cell exhaustion (49–53). However, donor-reactive CD4+ T cells expressing TIGIT were shown to decline in patients after kidney transplantation, suggesting a still unclarified role in geriatric patients after SOT (54). Interestingly, T cell exhaustion seems to be beneficial for outcomes after transplantation, as shown by Fribourg et al. in kidney transplant recipients (55). Immunosenescence also affects B cell maturation and antibody production, which negatively impacts humoral immune responses. Aging is associated with a distinct phenotype of B cells known as age-associated B cells, exhibiting a CD21−Tbet+ CD11c+ profile (56).

Important to note is the role of regulatory T-cells (Tregs) and B-cells (Bregs) which play cirtical role in maintaining immune tolerance and controlling excessive immune activation (57). With aging, the frequency and suppressive function of Tregs often increase, reflecting a compensatory response to the heightened inflammatory environment characteristic of immunosenescence. While elevated Treg activity may protect against autoimmunity, it can also impair effective immune responses to infections and malignancies, contributing to the immunodeficient phenotype observed in elderly individuals (58). Similarly, Breg populations, which secrete immunosuppressive cytokines such as IL-10, are dysregulated with age, leading to alterations in humoral immunity and further dampening of protective immune responses (59). In the context of transplantation, the expansion of Tregs and Bregs in older recipients may contribute to a lower incidence of acute rejection, but may also impair pathogen clearance and increase susceptibility to opportunistic infections, complicating post-transplant management strategies in the geriatric population.

The pathophysiological changes observed in older patients account for many of the differences in SOT outcomes and surgical management compared to younger patients. Although geriatric SOT patients with reduced B cell activity and decreased T cell function may experience heightened susceptibility to infections and malignancies, a decreased likelihood of acute rejection following organ transplantation is well described (17, 60–63). Geriatric patients often receive lower doses of immunosuppressive medications than their younger counterparts due to their less resilient immune systems. A study by Onaca et al. examined this observation in liver transplant recipients and found that patients younger than 60 had acute cellular rejection rates as high as 39.2%, while patients aged 60 and over had lower incidences at 28.8% (64, 65). The lower incidence of acute cellular rejection observed in older patients and the consequently reduced need for immunosuppressive medications help mitigate age-related morbidity and mortality that may otherwise occur due to rejection following SOT.

#### Innate immunity

**Figure 1:**
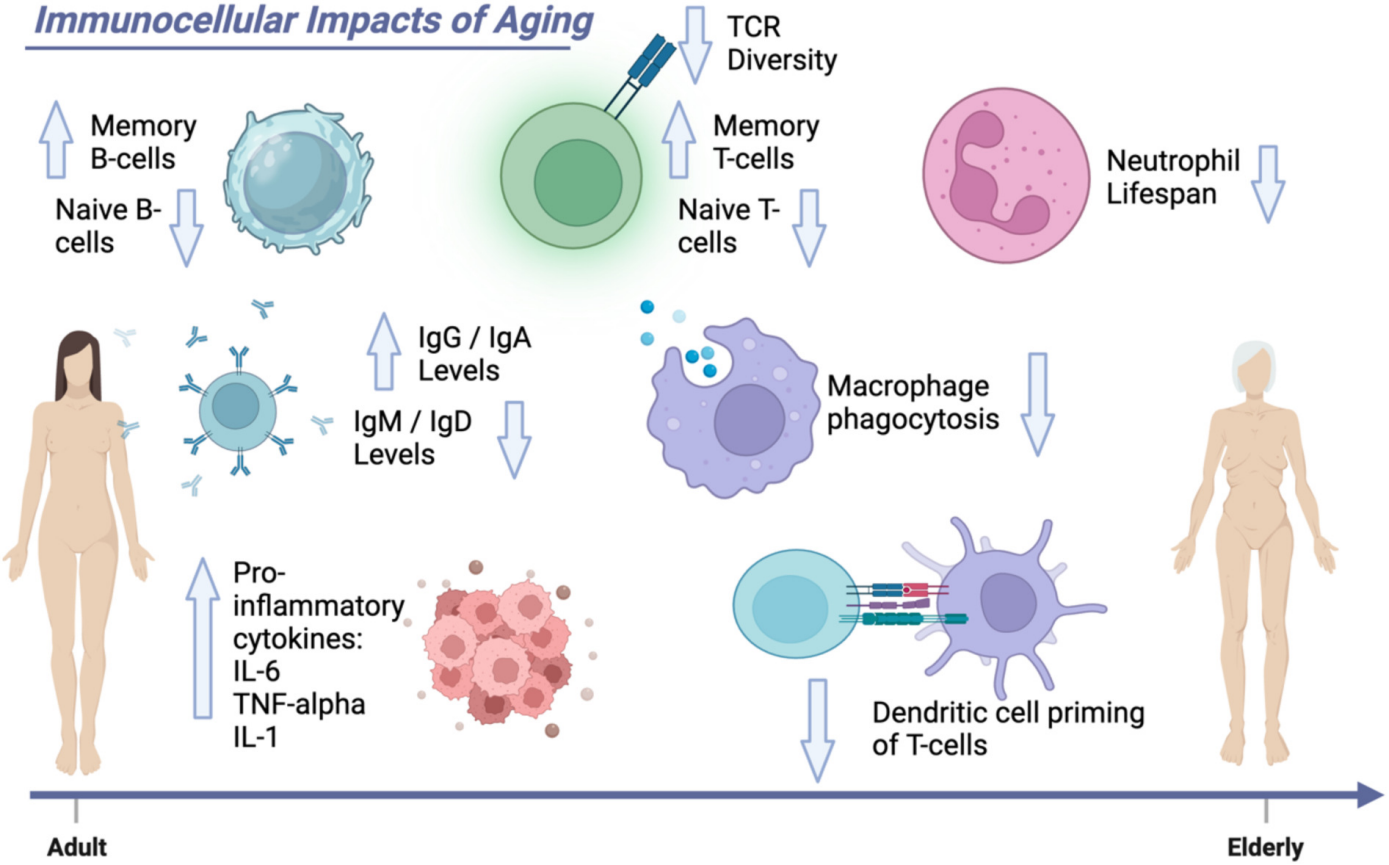
Immunocellular impacts of aging. This Figure demonstrates the age-related changes to the immune system. Elderly patients have decreased strength of their comprehensive immunity with decreased effectiveness of macrophage phagocytosis, decreased ability of dendritic cells to prime T cells, and decreased neutrophil lifespan.

Immunosenescence impacts not only adaptive immunity but also the innate immune system, resulting in functional impairments that alter host defense in elderly transplant recipients. Antigen-presenting and dendritic cells may experience limitations in their capacity to stimulate T cells and overcome tissue barriers in elderly patients with chronic illness due to dysregulation of IL-12 and IL-10 levels (66). These cytokines, produced by both antigen-presenting and dendritic cells, have antagonistically opposing functions and are known to normally mediate stimulation of T cells in adaptive immunity (67–70). In fact, this state has been referred to as senescence-associated secretory phenotype (SASP) which specifically references the pro-inflammatory and tissue-remodeling factors secreted by senescent cells, including cytokines, chemokines, growth factors, and proteases (71). As people age, the increased exposure to pathogens leads to a state where memory cells become overly adapted, rendering the elderly immune system more susceptible to new pathogens and inducing a chronic, irreversibly inflamed condition known as “inflammaging” (72, 73). Ultimately, inflammaging represents an accumulation of senescent cells and the corresponding SASP. Although it remains an area of active research, inflammaging may arise as a consequence of immune system restructuring where immune cells, such as macrophages, increase their secretion of pro-inflammatory cytokines and subsequently produce a more chronic state of inflammation in elderly patients (72, 73). When immunosenescence diminishes the effectiveness of the adaptive immune response, the body increasingly relies on inflammaging to safeguard against pathogens through the innate immune response. This enables a more rapid combatting of pathogens due to the constant activation of inflammatory reactions through cytokines commonly elevated in inflammaging (43) (Figure 1).

#### Impacts of immunosuppression medications

In clinical practice, immunosuppression is modified to ensure that the patient receives enough immunosuppression to avoid rejection, but not so much that they develop infections or an overly heightened risk of malignancy (74). Applying the complex cellular and molecular changes of aging to clinical practice requires 1. utilizing age-adjusted dosing of immunosuppressants, 2. incorporating frailty assessments into pre-transplant evaluations and post-transplant care plans to tailor interventions and identify high-risk patients, and 3. prioritizing proactive infection prevention strategies, such as vaccinations and close monitoring, given the immunosenescence associated with aging.

However, the adverse effects of immunosuppressant medication should not be discounted especially in elderly patient populations. The standard immunosuppressive regimens typically involve a combination of a calcineurin inhibitor (CNI) such as tacrolimus or cyclosporine, an antiproliferative agent like mycophenolate mofetil (MMF) or azathioprine, and corticosteroids (75). Although, mTOR inhibitors (e.g., sirolimus or everolimus) or co-stimulation blockers like belatacept may also be effectively utilized into maintenance therapy. Calcineurin inhibitors are cornerstone agents in most transplant protocols but are well known for their nephrotoxicity (76, 77). CNIs induce renal vasoconstriction, leading to chronic hypoperfusion, tubular atrophy, and interstitial fibrosis, contributing to chronic kidney disease over time. In elderly patients, whose baseline renal function may already be compromised due to aging-related nephrosclerosis, the risk of CNI-induced nephrotoxicity is magnified, often leading to progressive renal dysfunction post-transplant. Moreover, CNIs are associated with neurotoxic effects such as tremors, headaches, confusion, and, in severe cases, seizures, which may be misattributed to cognitive decline in older adults, delaying diagnosis and intervention. Antiproliferative agents like mycophenolate mofetil and azathioprine primarily target lymphocyte proliferation but can cause significant hematologic toxicities, including leukopenia, anemia, and thrombocytopenia (78). These cytopenias heighten susceptibility to opportunistic infections and impair wound healing—issues particularly concerning in geriatric patients who are already at higher risk for infection due to immunosenescence. Gastrointestinal side effects, notably diarrhea, nausea, and abdominal pain, are common with MMF and can exacerbate frailty and nutritional compromise in elderly patients, increasing the risk of sarcopenia and functional decline. Moreover, the risk of bone marrow suppression necessitates careful, frequent monitoring of complete blood counts, with dose adjustments tailored more cautiously in older patients.

Corticosteroids, often used as part of induction or maintenance immunosuppression, present a wide array of adverse effects that are acutely detrimental to geriatric recipients (79, 80). Steroid-induced hyperglycemia can precipitate or exacerbate diabetes mellitus, a major cause of morbidity and mortality post-transplant. Osteoporosis is another major concern, as corticosteroids accelerate bone resorption and inhibit bone formation, dramatically increasing the risk of fragility fractures in an already osteopenic elderly population. Neuropsychiatric side effects such as steroid-induced mood disturbances, psychosis, or cognitive impairment can further impair quality of life and complicate postoperative recovery in older adults. Additionally, long-term steroid use contributes to muscle wasting (steroid myopathy), further compounding frailty and impairing rehabilitation efforts post-transplant.

### Translational challenges in aging patients—from bench to biomarkers

As previously outlined, increasing age is generally associated with comorbidity, frailty, and a weakened immune system. Decreased immune system activity renders geriatric (transplant) patients more susceptible to environmental and internal stress (81, 82). Rejection is one of the most common and serious complications of transplantations. Therefore, translational research is investigatingreliable markers and parameters that can early indicate potential complications and detect possible rejection by the immune system (83).

### Troponin T

Many SOT postoperative complications are more prevalent in geriatric patients compared to their younger counterparts and, therefore, require age-conscious risk assessments that consider relevant biomarkers to gauge surgical outcomes. In the context of kidney transplants, older patients that are initially denied transplantation on the basis of risk assessment are likely to present with troponin T levels that are 45% more elevated compared to patients that are initially listed or deferred for the same transplantation (84). Further, research has shown that its elevation prior to transplant surgery can be predictive of perioperative cardiovascular risk and mortality following the transplantation procedure (85). Although some measures of surgical outcome risks already factor troponin C levels into their determination, it still has not been implemented into standardized preoperative testing (84).

### Additional biomarkers

In addition to elevated troponin T levels as indicators of poor postoperative outcomes, numerous other factors are currently under investigation to screen the patient's eligibility for transplantation (84). For example, Li and Lan compared different damage-associated molecular patterns (DAMPs) in terms of immune signals during transplantations. DAMPs are molecules that are released under conditions of significant cellular stress or tissue injury, including HMGB1 and S100 (79). HMGB1 is involved in transcriptional regulation and is produced in almost all nucleated cells of the body (86). It is released due to cell death or in response to stress and is often triggered by the release of TNF or IL-1 (87). HMGB1 regulates inflammatory responses and indirectly activates and stimulates innate immune cells. Interestingly, HMGB1 has been demonstrated to be more upregulated in elderly transplant patients (83). S100 is an important calcium-binding protein that regulates inflammation, cell differentiation, proliferation, and energy metabolism (88). The production of S100 protein increases in the early stage of rejection, which may serve as a potential biomarker in transplantation. Rodent models have suggested that this increase was more pronounced in aging animals (89). While these findings warrant in-human studies, S100 may serve as another clinically relevant biomarker to monitor geriatric patients following SOT.

### Targeted immunotherapies

In addition to biomarkers to predict postoperative outcomes, there are current clinical trial medications for kidney transplantations and DAMPS control that are considered to be stage 1/2 FDA-approved targeted drugs, which include lulizumab pegol, isatuximab-IRFC, and pegcetacoplan (83). Lulizumab is an antibody that binds to CD28 on the surface of T cells and prevents their activation when engaged by an antigen-presenting cell (90–92). It demonstrates favorable pharmacological characteristics, including high bioavailability, and has good tolerance across different dosage levels, with headache as the most common side effect (93). Of relevance, inhibition of the interplay between dendritic cells and T cells via CD28 has been shown to display age-dependent differences in efficiency. Isatuximab-IRFC is an anti-CD38 monoclonal antibody that has been initially used for the treatment of multiple myeloma, and may hold potential for immunotherapy in transplantation surgery due to its impact on plasma cell proliferation and increase in immunoglobulins (88, 89, 94). Isatuximab has been used to desensitize kidney transplant candidates since it can decrease anti-HLA antibodies and plasma cells that produce alloantibodies (95). Pegcetacoplan is a targeted complement inhibitor that binds C3 and its fragment C3b to block both C3 and C5 convertase activity. By halting C3 cleavage, it prevents downstream formation of C3a/C3b and the membrane attack complex, thereby mitigating complement-mediated transplant injury. In SOT, this mechanism may protect allografts from antibody-mediated rejection and recurrent complement-driven transplant failure. For example, in kidney transplants with recurrent C3-glomerulopathy or immune-complex-mediated membranoproliferative glomerulonephritis, clinical trials showed that pegcetacoplan markedly reduced glomerular C3 deposition and proteinuria while stabilizing renal function. These findings suggest pegcetacoplan could improve SOT outcomes by suppressing complement-driven inflammation in solid-organ transplantation. Given that older patients tend to have milder immune responses following SOT compared to their younger counterparts, lower dosages of lulizumab, pegcetacoplan and isatuximab might be administered for effective immunosuppression in older patients. Strategically employing both induction and maintenance immunosuppressants at lower, carefully titrated doses in geriatric transplant recipients may offer a beneficial balance, promoting graft acceptance while mitigating the heightened risk of infection associated with this vulnerable population.

### Organ specific clinical considerations and care in geriatric patients

#### Heart

Heart transplantation in older adults brings forward notable challenges that are particularly shaped by the aging cardiovascular system and the greater history of underlying cardiac disease. Preoperative imaging with chest computed tomography angiography (CTA) or transesophageal echocardiography should be standard in geriatric candidates to identify aortic atheroma and calcifications which occur in increased frequency with age (96). If significant aortic disease is detected, surgical strategies must be modified—such as selecting axillary or femoral arterial cannulation sites during cardiopulmonary bypass—to reduce the risk of stroke from embolic debris. Additionally, one of the most serious concerns for elderly heart transplant recipients is the risk of primary graft dysfunction (PGD), which remains a leading cause of early post-transplant mortality (97). The aged myocardium's reduced resilience, coupled with endothelial dysfunction and heightened vulnerability to ischemia-reperfusion injury, magnifies this risk. These concerns necessitate nuanced intraoperative management that focuses on gentle reperfusion techniques, maintaining controlled hemodynamic parameters, and utilizing vasodilators judiciously to avoid reperfusion injury. Postoperatively, clinicians should adopt a lower threshold for mechanical circulatory support (such as intra-aortic balloon pumps or temporary ventricular assist devices) if early signs of graft dysfunction appear, as timely intervention is key to survival (98). In addition, these concerns highlight the necessity of careful donor selection. For geriatric patients, ideal donor hearts have minimal ischemic time and preserved ventricular function. Specifically, clinical protocols should discourage the use of marginal or extended criteria donors in this population, as the aged myocardium has limited tolerance for additional ischemic stress (99). During procurement and implantation, minimizing cold ischemia time and optimizing preservation strategies (e.g., ex vivo perfusion systems when available) can be critical interventions to protect the fragile graft (100). Another organ-specific concern is cardiac allograft vasculopathy (CAV), a form of chronic rejection that is notably aggressive in older recipients (101). Pre-existing systemic endothelial dysfunction and accelerated atherosclerosis contribute to the rapid development of CAV. Routine surveillance with intravascular ultrasound (IVUS) or optical coherence tomography (OCT) should be integrated earlier and more frequently into post-transplant follow-up for geriatric patients (102). In addition, prophylactic strategies, including the early use of statins and antiplatelet therapy, have shown promise in slowing disease progression. Some transplant centers favor immunosuppression regimens incorporating mTOR inhibitors, such as everolimus, which may provide added protection against CAV when compared to calcineurin inhibitor-based protocols alone (103).

#### Kidney

Kidney transplantation in geriatric patients presents a distinct set of challenges. Similar to heart transplants above, age-associated endothelial dysfunction and increased susceptibility to ischemia-reperfusion injury make older kidneys—particularly when sourced from expanded criteria donors—less tolerant of ischemic stress. Clinical management must prioritize strategies to reduce cold ischemia time, including the preferential use of local donors and the use of machine perfusion preservation when available (104). Early postoperative interventions for elderly patients should involve aggressive volume management to optimize renal perfusion and vigilant monitoring for early signs of non-function, with a low threshold for initiating dialysis if oliguric states persist, to avoid volume overload and graft injury (105). Another significant concern in geriatric patients is the higher rate of chronic allograft nephropathy (CAN) and accelerated graft fibrosis. As a result, long-term care must focus not just on rejection surveillance but on early identification of chronic injury (106). Protocol biopsies at set intervals post-transplant may be particularly useful in elderly recipients to detect subclinical fibrosis and guide immunosuppressive adjustments before irreversible damage ensues. Accordingly, immunosuppressive management in elderly kidney transplant recipients must be specifically tailored to minimize nephrotoxicity, as these patients already exhibit reduced baseline nephron reserve and impaired regenerative capacity. Calcineurin inhibitors (e.g., tacrolimus, cyclosporine) accelerate chronic kidney damage and exacerbate age-related nephrosclerosis. Dose minimization strategies, combined with the early introduction of mycophenolate mofetil and consideration of belatacept-based regimens (where feasible), are particularly advantageous in preserving long-term graft function (107). Furthermore, corticosteroid maintenance, if needed, must be cautiously balanced against the risks of exacerbating post-transplant diabetes mellitus, which has a disproportionately detrimental effect on graft survival in older adults. Urological complications also carry special significance in geriatric kidney recipients. Bladder dysfunction, whether due to longstanding anuria or age-related detrusor instability, predisposes to urinary leaks, vesicoureteral reflux, and infections post-transplant (108). Preoperative urodynamic studies should be strongly considered in elderly candidates with histories of bladder dysfunction, lower urinary tract symptoms, or prolonged dialysis dependence. Tailored surgical modifications, such as ureteral stenting or even ureteral reimplantation techniques, may be necessary based on pre-transplant bladder evaluation findings (109).

### Survival and quality of life

In the case of end-stage renal disease, dialysis and kidney transplantation must be weighed up, especially in terms of the risks of surgery vs. gains in life expectancy and quality of life (QoL). At its core, kidney transplants in any group must yield better results than keeping patients on dialysis. Various studies have examined transplant outcomes in older individuals, comparing survival rates on hemodialysis to those following a transplant. Results from the Scientific Registry of Transplant Recipients revealed that transplant recipients aged 70 or older have a 41% reduced risk of death compared to those on the waiting list. Kidney transplantation has been shown to enhance life expectancy across all adult age categories, including individuals aged 70 and above, and this benefit persists regardless of how long patients wait before receiving a transplant. Notably, even organs donated by individuals aged 80 or older lead to improved survival compared to continuing dialysis treatment. In the U.S., data revealed that adults aged 75 and older who received kidneys from living donors had a five-year survival rate of nearly 60%, while those receiving organs from deceased donors had a 40% rate. In contrast, individuals of the same age group who were still waiting for a transplant had a survival rate of just under 30%, and those who were ineligible or not selected for transplantation had a rate as low as 12.5%. Already in the 1990s results from the from the U.S. Renal Data System pointed out a risk reduction for mortality 18 month after transplantation in patients 60–74 years old compared to patients still on the waiting list. Despite the risks associated with surgery, the risk to die returns to that of the reference population after less than six months (148 days). More recently published data reported a survival advantage after nine months post transplantation compared to patients on dialysis.

Several cohort studies have shown that older adults who undergo transplantation report better physical functioning and higher QoL than those who remain on dialysis. Additionally, QoL also improved in patients compared before and after kidney transplantation. These data suggest that for many elderly patients, kidney transplantation not only extends survival but also enhances day-to-day well-being and functional status, which are often of primary importance in this age group. As such, QoL outcomes should be a central consideration in treatment decision-making for older patients with end-stage renal disease. However, it must also be taken into account that not every patient with an indication for dialysis is eligible for surgery or can be guaranteed adequate follow-up care after transplantation. Furthermore, overall survival is also significantly influenced by comorbidities. In order to benefit from improved quality of life and longer survival as shown in the cited literature through kidney transplantation, potential recipients must be carefully evaluated, which should also include a geriatric assessment.

#### Liver

One of the foremost concerns with liver transplantation in elderly patients is the heightened risk of perioperative hemodynamic instability due to impaired cardiovascular reserve. Cirrhotic cardiomyopathy, often subclinical in younger patients, becomes more clinically significant in elderly candidates, who exhibit blunted contractile responses to stress, diastolic dysfunction, and increased systemic vascular resistance (110, 111). Preoperative cardiac evaluation must therefore go beyond standard ejection fraction measurement, incorporating stress echocardiography or even right heart catheterization to assess pulmonary pressures and cardiac output reserve (112). Intraoperatively, meticulous hemodynamic management, including vasopressor support tailored to maintain perfusion without inducing afterload stress, is essential during both the anhepatic and reperfusion phases. Older liver transplant recipients are also disproportionately prone to biliary complications, such as ischemic cholangiopathy and anastomotic strictures, primarily due to compromised hepatic arterial blood flow and microvascular disease inherent to aging (113). Surgical teams must prioritize careful donor-recipient arterial size matching, utilize meticulous microvascular technique, and consider arterial reconstruction methods when discrepancies are present. Postoperatively, early Doppler ultrasonography should be employed routinely within the first 24 h and at serial intervals to detect hepatic artery thrombosis or stenosis, with prompt endovascular or surgical intervention as needed to preserve biliary integrity (114). The risk of recurrent hepatocellular carcinoma (HCC) after transplant is another liver-specific challenge in the elderly, particularly as older candidates increasingly undergo transplantation for malignancy rather than for end-stage liver failure alone (115). Clinical protocols must ensure stringent selection criteria, strictly adhering to or being even more restrictive than the Milan criteria. Post-transplant, surveillance for HCC recurrence with cross-sectional imaging and alpha-fetoprotein (AFP) measurement should continue at regular intervals for at least five years, as delayed recurrences are not uncommon in older recipients.

#### Lungs

Older lung transplant recipients have a markedly increased risk of chronic lung allograft dysfunction (CLAD), particularly bronchiolitis obliterans syndrome (BOS) (116, 117). The pathogenesis of BOS is accelerated in the elderly due to age-related dysregulation of innate immune responses and impaired tissue repair mechanisms as outlined previously. Clinically, this necessitates a more aggressive and earlier surveillance strategy. Routine spirometry should be initiated immediately after discharge and repeated at least monthly for the first year post-transplant to detect early decline in forced expiratory volume (FEV1) (118). Any drop greater than 10% from baseline should prompt expedited evaluation, including bronchoscopy with transbronchial biopsy to diagnose and treat acute rejection or infection before irreversible airway remodeling occurs (119). Another critical lung-transplant-specific challenge is the heightened risk of restrictive allograft syndrome (RAS), a variant of CLAD characterized by fibrotic changes in the transplanted lungs (120). Elderly recipients seem disproportionately prone to RAS, likely due to reduced lung compliance and exaggerated fibroproliferative responses. Strategies to prevent RAS include early institution of azithromycin therapy (which has anti-fibrotic properties) and consideration of low-dose maintenance corticosteroids beyond standard tapering protocols. Infection risk is particularly devastating in lung transplant patients because the allograft is directly exposed to the external environment. Elderly recipients, with their reduced mucociliary clearance and diminished cough reflex, are especially susceptible to opportunistic pulmonary infections (121). Clinical care must therefore incorporate intensified antimicrobial prophylaxis, including lifelong Pneumocystis jirovecii pneumonia (PJP) prophylaxis, prolonged cytomegalovirus (CMV) prophylaxis, and broad antifungal prophylaxis, particularly against Aspergillus species (122). Vaccination protocols must be fully optimized prior to transplant, including pneumococcal conjugate and polysaccharide vaccines, annual influenza vaccination, and consideration of RSV prophylaxis during peak seasons. Postoperatively, aggressive bronchoscopy surveillance with routine bronchoalveolar lavage cultures is essential for early pathogen detection and preemptive therapy, as geriatric patients often manifest atypical or blunted signs of infection. Pulmonary vascular complications, including primary pulmonary hypertension recurrence and reperfusion pulmonary edema, are also more prominent in the elderly (123). Elderly patients with pre-existing pulmonary hypertension require preoperative optimization with pulmonary vasodilators and perioperative hemodynamic support using agents such as inhaled nitric oxide or phosphodiesterase inhibitors. Right heart catheterization should be used liberally post-transplant to guide management, as the right ventricle in older patients may have limited ability to recover from perioperative strain.

### Perioperative treatment strategies in elderly transplant patients

The assessment of risk factors during the preoperative stage of SOT is essential for geriatric patients. Preoperative assessment and optimization of comorbidities are important and can take place through various means (124). Although the Comprehensive Geriatric Assessment was not specifically developed for transplant surgery, it encompasses psychological, nutritional, and cognitive approaches, along with medication therapy adjustments tailored to elderly individuals. By integrating various individual needs, this assessment has the potential to improve long-term success for geriatric patients undergoing transplant surgery (125).

Kao et al., who compared different methods for measuring frailty, emphasized the potential of utilizing a preoperative risk assessment for improving patient care. They critiqued the lack of standardized assessment models used for SOT candidates and stressed the necessity for an individualized system for transplant surgery. With regards to age-related candidacy for SOT, they advocated for developing a more standardized approach to quantifying frailty and incorporating this score into risk assessments (19). A 2018 scoping review showed that 89 different measures were being used to quantify frailty in an acute care setting, with the most common tools including the Clinical Frailty Scale, the Frailty Index, and the Frailty Phenotype. Kao et al. felt they should be consolidated and made relevant in the context of SOT (19, 126). In addition to subjective estimations, it is now possible to incorporate laboratory testing into preoperative assessment scales. Haugen et al. discovered that IL-6 indicated an increased postoperative mortality risk in up to 40% of kidney transplant patients, and that measuring such laboratory markers in combination with frailty provided more insight into the surgical outcome than measuring inflammatory indices alone (127). Miura et al. examined a risk assessment tool for preoperative surgery in elderly patients, analyzing the influence of various factors on postoperative outcome quality.

In terms of intraoperative implications, differences in pharmacokinetics and pharmacodynamics among patients from different age groups require that anesthetic management be tailored to the geriatric population. Although patients over the age of 65 undergoing SOT may not have any underlying health conditions, their likelihood to exhibit distinct pharmacological responses to anesthesia compared to younger patients often necessitates that they be administered lower dose regimens. By 95 years of age, the half-life of a given anesthetic is usually twice the value that is typically observed in younger adults due to decreased renal function (128). Despite receiving reduced medication to mitigate the age-related changes in pharmacokinetics, the effects of these lower dosages often endure longer than anticipated. Such observations reflect the importance of factoring the unique medical histories and risk considerations of geriatric patients into their anesthetic management during SOT (36, 125).

Recovery is a complex and gradual process that does not simply commence after a surgical procedure, but also encompasses the initial phases that occur intraoperatively. Geriatric patients may experience a slower recovery trajectory and face an augmented risk of postoperative complications, including delirium, infections, and functional decline (please see “Age-Specific Clinical Considerations and Common Risk Factors of Postoperative Complications”). For geriatric patients, wound healing tends to be delayed and less effective, necessitating a protracted wound care regimen. Age-related prolonged wound healing can be attributed to an increase in platelets that adhere to damaged epithelium and subsequently clot in older patients. The resulting clot and surrounding damaged tissue release both pro-inflammatory cytokines and growth factors. These agents, such as TGF-β, PDGF and EGF facilitate inflammation and ultimately allow for the infiltration of neutrophils and monocytes to the wound site in order to further facilitate repair (129, 130). Although monocytes generally rely on the expression of adhesion molecules to localize to the site of injury, these molecules are less abundant in aged skin and therefore impede recruited monocytes from adhering and maturing into macrophages to facilitate wound repair (131–134). It is therefore advisable to incorporate additional healing-promoting treatments (e.g., acellular allograft dermis) and monitoring specifically tailored to elderly patients (135, 136).

**Figure 2:**
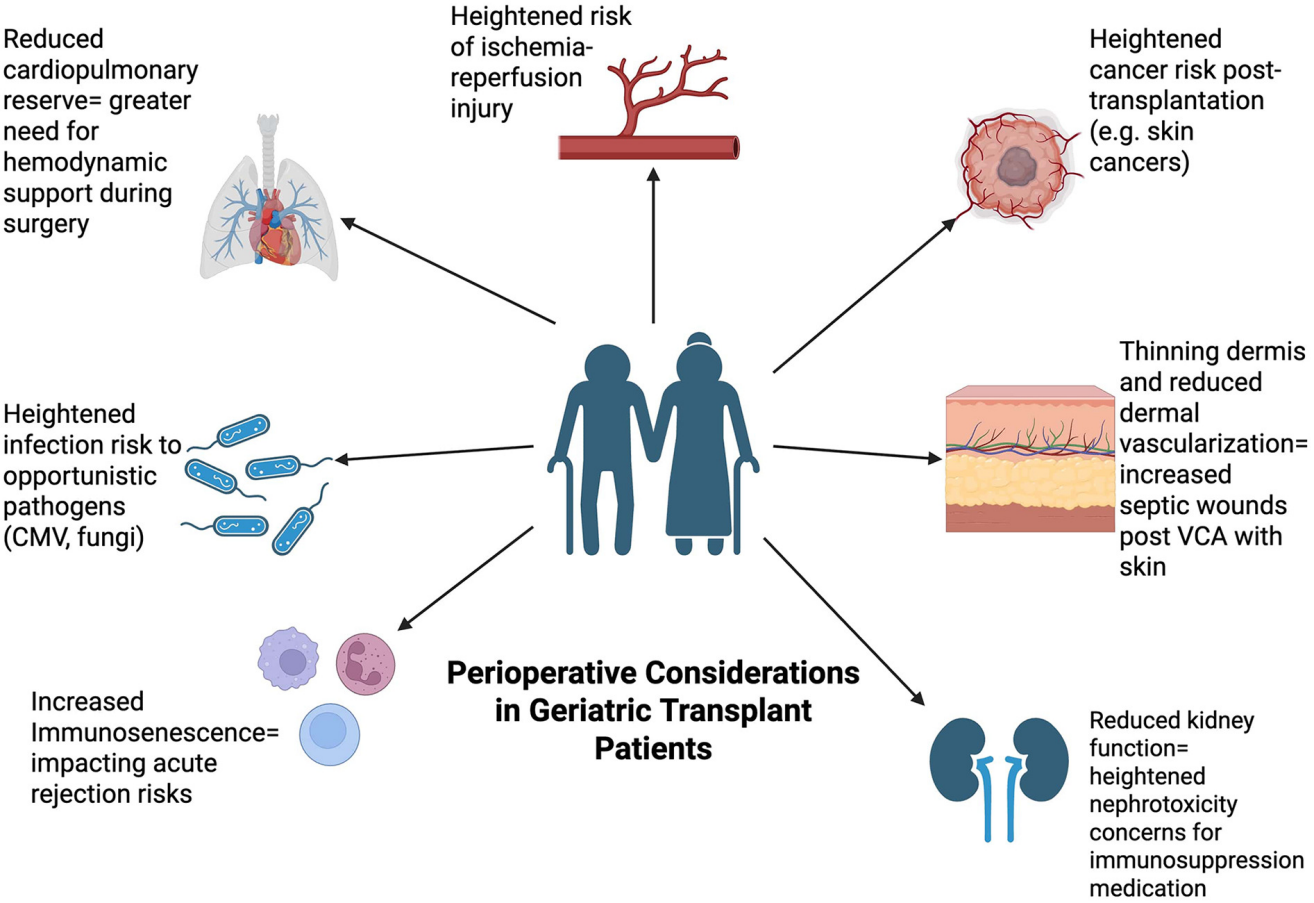
Perioperative considerations in geriatric patients. This Figure depicts the different perioperative considerations that are relevant in the context of solid organ transplantation surgery within the geriatric population. Preoperative factors include prehabilitation and muscle mass while intra-operative factors include anesthesia dosages due to impaired metabolism. Postoperative factors include managing immunosuppression medication due to reduced immune system as well as delayed wound healing, bleeding risk, and delirium.

Postoperative care of geriatric patients can also be improved following SOT through the implementation of skilled nursing facilities. The demand for such facilities is increasingly greater for patients with increased frailty after SOT compared to geriatric patients presenting with less frailty. A study on kidney transplantation recognized the specific need for rehabilitation in geriatric patients. It was observed that geriatric patients with a higher Frailty Risk Score were rehospitalized an average of 2.9 times more often than patients with a lower score (137). Such outcomes have the potential to be reduced through continued care at a skilled facility. Following SOT, 40.9% of patients 70 years and older are discharged to a skilled nursing facility compared to only 15.9% of younger patients. These posthospitalization facilities differ from nursing homes in that they continue to include healthcare professionals that operate under the supervision of a doctor so that patients can ultimately transition to returning home (138) (Figure 2).

## Vascularized composite allotransplantation

### Elderly patients in VCA surgery

VCA surgery represents an emerging branch of SOT, integrating core concepts of organ transplantation, and thus, knowledge on elderly SOT patients may partially translate into this field. However, the unique tissue heterogenicity continues to challenge VCA providers, especially in elderly patients (139). To date, VCA surgery is commonly recommended in younger patients (less than 65 years). In fact, the oldest VCA recipient to undergo transplant surgery was 64-year-old Canadian, who received a face transplant in 2018. However, recent strides in immunosuppressive regimens may pave the way to provide VCA care to elderly patients. While comprehensive research on VCA outcomes for geriatric patients is scarce, the different VCA tissue types have been separately investigated in geriatric patients.

For example, histological shifts in the aging dermis and epidermis (e.g., collagen degradation, reduced dermal vascularization, decreased Langerhans cell counts, epidermal atrophy) may contribute to the gradient in VCA outcomes. Such shifts have been demonstrated to impact the transplantation of skin tissue, resulting in higher incidence of septic wounds and poor wound healing (i.e., low levels of growth factors such as PDGF, TGF-β, and VEGF) (140, 141). In humans, the oral mucosa demonstrates a loss of elastic fibers with corresponding disorganization and thickening of the collagen bundles underlying the connective tissue with aging, which, accompanied by a reduction in the microvasculature, can impede wound healing following trauma or insults to the mucosa (142–144). This phenomenon was exemplified by a cross-sectional evaluation of 750 geriatric adults by Cheruvathoor et al., where the authors found a significant decrease in collagen elasticity and microvasculature in patients over 65 years of age (145). From an immunological perspective, studies on rats by Santiago et al. have demonstrated that production of TGF-b and IL-10 in mucosal tissue of older mice was significantly reduced with aging, thus decreasing the ability of the dendritic class to stimulate TGF-β and differentiate CD4+ T cells (146). Similar functionality of these immunological species has been identified within human tissue as well, which is significant in VCA models as TGF-β and IL-10 serve as inhibitory cytokines that dampen the immune response to antigens and general pro-inflammatory mediators (147). Kauke-Navarro et al. have underscored the significant antigenicity of mucosal tissue following VCA surgery (10, 148, 149). Thus, a mitigation of these inhibitory cytokines can stimulate the pro-inflammatory environment within the mucosal tissue, and given its high antigenicity baseline characteristic, can result in potentially heightened risks of acute or chronic rejection in older patient populations.

Whilst VCA-focused studies on elderly patients are rare, aging patients demonstrated diverging outcomes following muscle and oseteocutaneous flap surgery. For example, Weaver et al. retrospectively reviewed 354 patients that underwent osteocutaneous free flap transfer and found that patients 70 years of age and older were 25% more likely to be discharged to skilled nursing facilities for posthospitalization care compared to younger patients (138).

Overall, these studies mainly investigated outcomes following single-tissue and/or autologous transplant surgery, limiting their generalizability. Based on these preliminary insights, future research on geriatric VCA patients is warranted to broaden access to VCA surgery in these age brackets.

## Conclusion

The global rise in the elderly population presents unique challenges to healthcare systems, particularly in specialized surgical fields like transplantation. This review examines the growing impact of the aging population on SOT and VCA. We discuss the increasing prevalence of geriatric patients undergoing these procedures, highlighting the unique needs and considerations associated with this demographic. Furthermore, we explore the current state of specialized care models for elderly transplant patients, emphasizing the need for further research and development of integrated, multidisciplinary approaches to optimize outcomes in this growing population. Accordingly, the authors strongly support the use of transplantation in geriatric patients, recognizing the significant improvements in survival and quality of life it can offer even in advanced age. Incorporating the special considerations outlined—ranging from adjustments in immunosuppressive strategies to proactive management of inflammaging and immunosenescence—is essential to optimize outcomes. Transplanting older adults is not simply an extension of traditional protocols but requires a tailored, biologically informed approach to achieve the best possible health and functional recovery for this vulnerable yet highly deserving population.
